# serocalculator, an R package for estimating seroincidence from cross-sectional serological data

**DOI:** 10.1101/2025.06.04.25328941

**Published:** 2025-10-06

**Authors:** Kristina W. Lai, Chris Orwa, Jessica C. Seidman, Denise O. Garrett, Samir K. Saha, Dipesh Tamrakar, Farah Naz Qamar, Richelle C. Charles, Jason R. Andrews, Peter Teunis, Kristen Aiemjoy, Douglas Ezra Morrison

**Affiliations:** 1. Department of Public Health Sciences, University of California Davis School of Medicine, Davis, CA, USA; 2. SkyeHi Technologies, Nairobi, Kenya; 3. Sabin Vaccine Institute, Washington, DC, USA; 4. Child Health Research Foundation, Dhaka, Bangladesh; 5. Dhulikhel Hospital, Kathmandu University Hospital, Dhulikhel, Nepal; 6. Department of Paediatrics and Child Health, Aga Khan University, Karachi, Pakistan; 7. Harvard Medical School, Harvard University, Boston, MA, USA; 8. Department of Immunology and Infectious Diseases, Harvard TH Chan School of Public Health, Harvard University, Boston, MA, USA; 9. Division of Infectious Diseases and Geographic Medicine, Stanford University School of Medicine, Stanford, CA, USA; 10. Hubert Department of Global Health, Center for Global Safe WASH, Rollins School of Public Health, Emory University, Atlanta, Georgia, USA; 11. Department of Immunology and Microbiology, Mahidol University Faculty of Tropical Medicine, Bangkok, Thailand

## Abstract

**Motivation:**

Seroincidence—the rate of new infections in a population—is a key measure for understanding pathogen transmission dynamics and informing public health action. Estimating seroincidence from cross-sectional data is complicated by antibody waning, cross-reactivity, and individual heterogeneity in antibody responses.

**Implementation:**

**serocalculator** is an open-source R package that uses a likelihood-based framework incorporating antibody decay models, biological variability, and measurement noise to estimate seroincidence rates from cross-sectional serological data.

**General features:**

The package supports overall and stratified seroincidence estimation using single or multiple biomarkers. It requires three inputs: (1) a pre-estimated seroresponse model characterizing post-infection antibody waning; (2) noise parameters capturing biological and assay-related variability; and (3) quantitative antibody responses from a cross-sectional survey. It is computationally efficient, well-documented, and includes a point-and-click R Shiny interface. These features promote usability across research and public health.

**Availability:**

The package **serocalculator** is freely available on CRAN, with development versions on GitHub.

## Introduction

Serological surveys can be used to measure population-level antibody responses to characterize infection occurrence (1,2). Following pathogen exposure, the adaptive immune system generates antigen-specific antibodies, which increase rapidly after infection and gradually wane over time (1,3,4). The concentration of antibodies against a given pathogen reflects the time since last infection (2–4). In cross-sectional surveys, a large proportion of individuals with high antibody concentrations suggests high burden of recent or frequent infection, while a smaller proportion indicates lower burden.

Population-level antibody responses are typically characterized using two key epidemiological measures: seroprevalence, the proportion of individuals with antibody levels above a defined threshold; and seroincidence, the rate at which new infections occur in the population (1,2). The magnitude and speed of an individual’s immune response to infection is complex and dynamic, shaped by infectious dose, age, disease severity, prior exposures, and vaccination history. Most analytic methods and software used to estimate seroprevalence and seroincidence dichotomize quantitative antibody levels into binary outcomes—seropositive or seronegative. While simple and widely used, this approach ignores temporal changes in antibody concentrations, individual-level heterogeneity, and immunological cross-reactivity between pathogens. Moreover, seroprevalence reflects the cumulative history of prior exposures in a population and is influenced by the age distribution of the sample, making it an imperfect proxy for recent transmission. In contrast, seroincidence rates quantify the rate at which new infections occur and are increasingly favored by epidemiologists, modelers, and public health professionals (3,4). These rates can help determine whether a disease has been eliminated (5,6), identify risk factors for transmission, and locate areas where interventions are most needed (3,7).

Traditional methods to assess seroincidence require resource- and time-intensive longitudinal studies that are often infeasible. However, new statistical methods estimate seroincidence rates from cross-sectional serosurveys by incorporating models of antibody decay dynamics from confirmed cases (2,8,9). Seroincidence can be estimated as a function of peak antibody response and decay rate from infected individuals while accounting for heterogeneity in immunity, responses to multiple antigens, age-dependence, non-specific binding of the assay probes (noise), and laboratory assay variability (1,8–10). This approach assumes that exposure follows a Poisson process, meaning all individuals in the sampled population are subject to a constant infection risk over time. Using this assumption, maximum likelihood estimation (MLE) is used to estimate seroincidence from cross-sectional serologic data (2,8–11). We also assume that seroresponses from each antibody isotype are independent, allowing us to sum the likelihoods from multiple antibodies and antigens, thereby increasing precision in the seroincidence estimate (1,9). Here, we introduce **serocalculator**, a statistical software package that offers a validated and accessible framework for estimating seroincidence rates from cross-sectional serosurveys by integrating antibody kinetics with noise correction to produce dynamic infection rate estimates (1,8–10). It includes intuitive tools such as multi-biomarker support, stratified analysis, and an R Shiny interface for non-coders. An earlier package, *seroincidence* (12), was developed and maintained from 2015–2018, but lacked methods for incorporating biologic and measurement noise, necessitating a full revision. The new **serocalculator** package supersedes *seroincidence* by incorporating the latest methodological advancements for seroincidence estimation.

### Implementation

**serocalculator** can be downloaded from the Comprehensive R Archive Network (CRAN). Over 30 exported functions guide users from data import and cleaning to analysis and visualization. All functions in **serocalculator** version 1.4.0 are described on the package website. The general workflow is: 1) import data, 2) inspect and visualize data, and 3) estimate seroincidence rate.

### Import Data

Three inputs are required: 1) Previously-estimated longitudinal seroresponse model parameters characterizing the longitudinal response of the surveyed serum antibodies; 2) Noise parameters that encompass biological noise (individual variation in response) and measurement noise (laboratory assay variability); and 3) Quantitative antibody levels from a cross-sectional population-based serosurvey sample that is calibrated on the same scale as the longitudinal seroresponse parameters. For testing purposes, users can access small example files within the package, or they can import larger sample files for specific diseases from the publicly available Serocalculator Data Repository on Open Science Framework. Example input datasets and formatting guidance are provided in Supplement S1.

#### Longitudinal Seroresponse Parameters

Longitudinal seroresponse parameters describe how antibodies rise and fall after infection. Before using **serocalculator** to estimate seroincidence, these parameters must be previously estimated by fitting two-phase, hierarchical within-host models to quantitative antibody responses measured longitudinally in a cohort of confirmed cases, using a Bayesian inferential framework and a Markov Chain Monte Carlo (MCMC) computational approach to sample from the joint predictive posterior distribution of the model parameters for each antigen isotype (1,8–10) (see Supplement S2). Seroresponse models can be stratified by age, country, and/or other relevant demographic characteristics, if seroresponse dynamics vary among the corresponding subpopulations.

Users can import their own set of seroresponse parameters in either CSV or RDS format and then apply as_sr_params(), which will assign the imported dataset as an sr_params object and ensure required variables are present and in the correct format. If using online files from a URL, users can apply the function load_sr_params(), which will perform necessary column and formatting checks. The longitudinal parameter sets must include the following columns: one or more disease-specific antigen and antibody isotype pairs that matches those included in the cross-sectional population data (antigen_iso), the baseline antibody concentration (yθ), the peak antibody concentration (y1), the time to peak antibody concentration (t1), the antibody decay rate (alpha), and the antibody decay shape (r).

Further details on the longitudinal seroresponse parameters can be found in Supplement S2.

#### Noise Parameters

The noise parameters object defines assumptions about the measurement process.(1) Biological noise (*v*) reflects potential non-specific antibody binding from other exposures, such as cross-reactivity with a related pathogen. It is defined as the 95th percentile of the antibody response distribution, assumed to be normal, in a population with no prior exposure to the pathogen.(1) Measurement noise (*ϵ*) represents laboratory assay variability from the laboratory testing process. It is defined by a coefficient of variation (CV), or the ratio of the standard deviation to the mean for replicates across plates. The **serocalculator** package can also accommodate censored data: lower and upper limits of reliable quantification may be specified as y_low_ and y_high_, respectively, and any observations recorded outside of those limits will be treated as censored.

As above, users can import their own noise parameters into R (e.g., from CSV or RDS files) as a data.frame and then apply as_noise_params(), which will convert the data.frame into a noise_params object (a subclass of tibble::tbl_df with added metadata attributes) and ensure required variables are present and in the correct format. If using online files from a URL, users can apply the function load_noise_params() and then follow the process described above for the seroresponse parameters. The required noise parameter variables are: one or more disease-specific antigen and antibody isotype that match those included in the cross-sectional population data (antigen_iso), the biological noise (nu), the measurement noise (eps), and the lower and upper limits of detection of the assay (y.low and y.high respectively). Further details on the noise parameter calculations are in Supplement S3.

#### Cross-sectional population data

Once again, users can import their own cross-sectional population data in either CSV or RDS format and apply the function as_pop_data(), which assigns the imported dataset as a pop_data object and ensures correct formatting. The required variables for this dataset are: age, measured in years (age), one or more specified antigen isotype pairs (antigen_iso), quantitative antibody response (value), and a unique participant id (id). The as_pop_data() function detects the required variables or allows users to specify them. If using online data from a URL for an online repository such as Open Science Framework, users can apply the function load_pop_data() and then once again follow the process described above for the seroresponse parameters. Population datasets can include additional variables for stratification.

### Estimating seroincidence

Once all parameters and datasets are imported, users can estimate either overall or stratified seroincidence rates using the functions est_seroincidence() and est_seroincidence_by(), respectively. Users begin by specifying the three required data inputs and selecting one or more antigen-isotype pairs for inclusion in the estimate. Optional parameters allow customization of visualizations, warning messages, parallel computing, and the estimation process. Stratified seroincidence estimates can be generated by specifying one or more covariates in the cross-sectional dataset, and optionally in the longitudinal seroresponse parameters.

### Use

We will demonstrate the use of **serocalculator** with a reproducible example of enteric fever seroincidence using a subset of data from a series of serosurveys conducted in Bangladesh, Nepal and Pakistan in 2016–2021 (13,14). Additional details on these data are available in Supplement S4. Data for this analysis are stored in the Serocalculator Data Repository on Open Science Framework. This example is also available as an article on the **serocalculator** website.

First, we load each of our three inputs (cross-sectional population data, noise parameters, and seroresponse parameters) and then create descriptive plots for both cross-sectional data and modeled seroresponse parameters using built-in autoplot() visualization methods. In this example, we import noise parameters calculated at each study site laboratory from analysis using enzyme-linked immunosorbent assays (ELISA).

**Figure 2:**
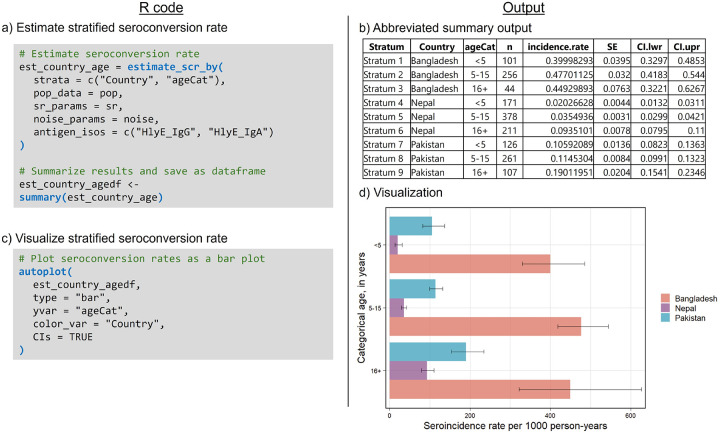
Estimation of seroincidence rates stratified by country and age category. a) Code for executing and summarizing results of the est_seroincidence_by() function. b) Output from summary() for stratified seroincidence estimates. c) Code for plotting seroincidence rate estimates by country and age category as a bar plot. d) Output from autoplot() for visualizing seroincidence by strata.

We can estimate overall and stratified seroincidence rates using the est_seroincidence() and est_seroincidence_by() functions, respectively. In both cases, we combine IgA and IgG seroresponses to the hemolysin E (HlyE) antigen (Figure 2a). For the overall seroincidence rate, we choose to filter to only sites in Pakistan and find a seroincidence rate of 128 new infections per 1000 person-years (95% CI). To compare across countries and ages, we specify the strata variables and find that 5–15-year-olds in Bangladesh experience the highest seroincidence of enteric fever, with a rate of 477 new infections per 1000 person-years (Figure 2). Model outputs from est_seroincidence() and est_seroincidence_by() can be summarized and visualized using the corresponding summary() and autoplot() methods, respectively.

## Discussion

**serocalculator** provides a rapid and computationally simple method for calculating population-level seroincidence rates that account for antibody decay dynamics, biologic variability, and measurement noise and can integrate information from multiple biomarkers. Building upon the prior *seroincidence* package (12), the ***serocalculator*** package implements updated analytic methods that account for biological and measurement noise, new function names, new summary and visualization options, and cross-sectional data simulations.

Several groups have developed R packages to support seroepidemiologic analysis. The *serosv* package estimates seroprevalence and force of infection (FOI) using built-in datasets, but it is not intended for user-supplied data (15). *serosim* simulates serological study data to support study design and evaluate within-host dynamics and new analytic approaches (16). While **serocalculator** also includes a simulation function, its primary purpose is to estimate seroincidence rates from user-collected data. *Rsero* provides Bayesian tools for comparing serocatalytic models, assessing FOI under various assumptions (e.g., transmission mode, seroreversion), and evaluating sampling strategies (17). *serojump* focuses on infection timing and antibody kinetics using Bayesian reversible jump MCMC and individual-level data (18). Collectively, these packages expand access to seroepidemiologic tools. Notably, **serocalculator** is the only package to incorporate individual-level heterogeneity, combine multiple antigen isotypes, and account for multiple sources of noise.

Future enhancements will address current methodological limitations and expand usability. While **serocalculator** supports interval censoring for some assays, its application is constrained by the availability of longitudinal seroresponse parameters for specific pathogens and assays. To increase flexibility across study settings, we are developing a complementary R package that will enable users to model seroresponse parameters from their own data.

Another key development area is improving performance in high-burden settings, where repeated exposures can bias seroincidence estimates by changing the distribution of longitudinal seroresponse patterns. Current methods do not fully address this challenge, and we are actively working to incorporate Approximate Bayesian Computation techniques to enhance estimation in areas with high force of infection (11).

The **serocalculator** package has been applied to studies of enteric fever and scrub typhus (14,19,20), and its methods are currently being adopted by research teams and public health institutions to inform vaccine introduction decisions and surveillance strategies in endemic regions. **serocalculator** provides a free, open-source, and user-friendly tool for estimating seroincidence rates from cross-sectional quantitative serology data. Additional documentation and tutorials are available at https://ucd-serg.github.io/serocalculator/.

## Supplementary Material

Supplement 1

Supplementary material are available online.

## Figures and Tables

**Figure 1:**
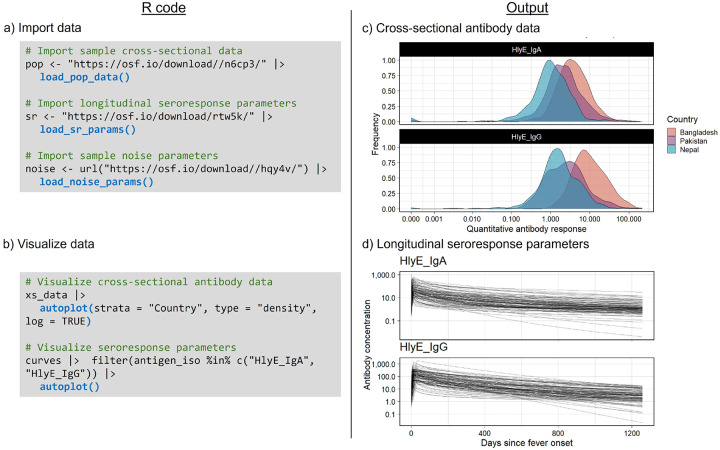
Code and visualization of log antibody responses from three study sites. a) Code for importing the three required inputs. b) Code for visualizing cross-sectional antibody data and longitudinal seroresponse. c) Plot for cross-sectional curve parameters. d) Plot for longitudinal seroresponse parameters.

## Data Availability

Source code for the **serocalculator** package is available on GitHub: https://github.com/UCD-SERG/serocalculator. Deidentified data from the sample analysis is hosted on Open Science Framework: https://osf.io/ne8pc/files/osfstorage.
