# Supplementary material for: serocalculator, an R package for estimating seroincidence from cross-sectional serological data
Source: medRxiv. 2025 Aug 4:2025.06.04.25328941. Originally published 2025 Jun 6. Preprint. [Version 2] doi: 10.1101/2025.06.04.25328941 (PMC12155044; doi:10.1101/2025.06.04.25328941)
Supplement: Supplementary file 1 [file NIHPP2025.06.04.25328941V2-supplement-1.pdf]

medRxiv preprint doi: https://doi.org/10.1101/2025.06.04.25328941; this version posted August 4, 2025. The copyright holder for this preprint (which was not certified by peer review) is the author/funder, who has granted medRxiv a license to display the preprint in perpetuity.
It is made available under a CC-BY 4.0 International license .

Supplementary material for: '**serocalculator**, an R package for estimating seroincidence from cross-sectional serological data'

Table of Contents

S1. Dataset Formatting Requirements 2
S2. Further Details on Longitudinal Seroresponse Parameters 4
S3. Further Details on Noise Parameters 9
S4. Description of Use Case Dataset 10

medRxiv preprint doi: https://doi.org/10.1101/2025.06.04.25328941; this version posted August 4, 2025. The copyright holder for this preprint (which was not certified by peer review) is the author/funder, who has granted medRxiv a license to display the preprint in perpetuity.
It is made available under a CC-BY 4.0 International license .

S1. Dataset Formatting Requirements

The table shells below are examples of formatted input datasets for each of the three required inputs for **serocalculator**. Any of the inputs may include variables to stratify by, but only strata in the cross-sectional population data will produce stratified seroincidence estimates. Noise parameters and longitudinal seroresponse parameters may be used by strata or as overall values applied equally to each cross-sectional strata.

Table S1.1 Cross-sectional population data

id	age	antigen_iso	value	strata1
1001		A		1
1001		B		1
1002		A		1
1002		B		1
1003		A		2
1003		B		2

- **id** = Unique identifier for each individual
- **age** = Numeric age in years (can include decimals)
- **antigen_iso** = Antigen and antibody isotype pair(s)
- **value** = Quantitative antibody response value
- **strata variables** (optional) = Any demographic features or covariate relevant to your analysis (must match those in other inputs to be used in analysis)

Table S1.2: Noise parameters

antigen_iso	y.low	y.high	eps	nu	strata1
A					1
A					2
B					1
B					2

- **antigen_iso** = Antigen and antibody isotype pair(s)
- **y.low** = Lower limit of detection of the assay used to produce the cross-sectional survey data set (a)
- **y.high** = Upper limit of detection of the assay
- **eps** = Measurement noise of the assay; CV%, range 0-1 (See Supplement S3)
- **nu** = Biologic noise of the assay; 95% of the distribution among those never exposed (See Supplement S3)
- **strata variables** (optional) = any demographic features or covariates relevant to your analysis (must match or be a subset of the stratifying variables included in the cross-sectional data)

medRxiv preprint doi: https://doi.org/10.1101/2025.06.04.25328941; this version posted August 4, 2025. The copyright holder for this preprint (which was not certified by peer review) is the author/funder, who has granted medRxiv a license to display the preprint in perpetuity.
It is made available under a CC-BY 4.0 International license .

Table S1.3: Longitudinal seroresponse parameters

antigen_iso	y0	y1	t1	alpha	r	strata1
A						1
A						2
B						1
B						2

- **antigen_iso** = Antigen and antibody isotype pair(s) measured
- **y0** = Baseline antibody concentration (U/mL)
- **y1** = Peak antibody concentration (U/mL)
- **t1** = Time to peak antibody concentration (days)
- **alpha** = Antibody decay rate (1/days)
- **r** = Antibody decay shape
- **strata variables** (optional) = any demographic features or covariate relevant to your analysis (must match or be a subset of the stratifying variables included in the cross-sectional data)

medRxiv preprint doi: https://doi.org/10.1101/2025.06.04.25328941; this version posted August 4, 2025. The copyright holder for this preprint (which was not certified by peer review) is the author/funder, who has granted medRxiv a license to display the preprint in perpetuity. It is made available under a CC-BY 4.0 International license .

S2. Further Details on Longitudinal Seroresponse Parameters

The longitudinal seroresponse parameters provided by the user in Table S1.1 are components of the within-host seroresponse model proposed in de Graaf et al. 2014 and extended in Teunis et al. 2016 and Diekmann et al. 2018.[1-3] The model consists of two phases: an active-infection/antibody growth phase and a post-infection/antibody decay phase. The active infection phase of the model is a modified version of the Lotka-Volterra model,[4,5] which is typically used to describe predator-prey dynamics in ecological systems. In **serocalculator**, this model is adapted to represent the competitive relationship between the host's adaptive immune system cells (predators) and the pathogen (prey), where the current rate of change in the concentration of each population depends on the current concentration of the other population. The general form of the Lotka-Volterra model is shown below:

Rate of change in Population X (prey): $x'(t) = \alpha x(t) - \beta x(t)y(t)$
Rate of change in Population Y (predator): $y'(t) = -\gamma y(t) + \delta x(t)y(t)$

Parameters:
$x(t)$ = size of prey population at time t
$y(t)$ = size of predator population at time t
α = prey's maximum per capita growth rate
β = effect of predators on prey death rate
γ = predator's per capita death rate
δ = effect of prey on predator's growth rate

The seroresponse model simplifies the Lotka-Volterra model to represent the immune response during the active infection period.[1,3] In the seroresponse model, the following differential equations represent the dynamics of pathogen growth, antibody production, and antibody decay (waning) after the end of the active infection, when the pathogen has been eliminated:

Pathogen growth during infection: $b'(t) = \mu_0 b(t) - cy(t)$
Antibody growth during infection: $y'(t) = \mu_1 y(t)$
Antibody decay after infection: $y'(t) = -\alpha y(t)^r$

Parameters:
$b(t)$ = pathogen concentration at time t
$y(t)$ = antibody concentration at time t
μ_0 = pathogen growth rate
μ_1 = antibody growth rate
c = rate of pathogen elimination
α = Antibody decay rate (1/days)
r = Antibody decay shape

medRxiv preprint doi: https://doi.org/10.1101/2025.06.04.25328941; this version posted August 4, 2025. The copyright holder for this preprint (which was not certified by peer review) is the author/funder, who has granted medRxiv a license to display the preprint in perpetuity.
It is made available under a CC-BY 4.0 International license .

The model switches from the antibody growth (active infection) phase to the antibody decay (post-infection) at the time point when the pathogen concentration $b(t)$ first reaches 0. This time point is denoted t_1, and can be calculated as:

$$t_1 = \frac{1}{\mu_1 - \mu_0} \log(1 + \frac{(\mu_1 - \mu_0)b(0)}{c\, y(0)}$$

During the growth phase, the antibody concentration grows exponentially and can be calculated for any given time t as:

$$y(t) = y(0)e^{\mu_1 t}$$

In the Teunis et al. 2016 model, the parameters $\mu_0, c, \mu_1, d,$ and r and initial conditions $b(0)$ and $y(0)$ are allowed to vary between individuals, and potentially between repeated infections of the same individuals. After recruiting a longitudinal cohort of infected individuals and collecting longitudinally-repeated blood specimens over time from each individual, we can estimate each participant's individual-specific parameters as well as the underlying population distributions of those parameters, using a multilevel (a.k.a. "hierarchical") random-effects longitudinal modeling framework[6] and a Bayesian inferential approach.[7]

The result of the longitudinal model is a two-phase response curve of antibody dynamics (Figure S2.1). Beginning at time t_0, or the initial time of infection, there is an initial exponential growth phase until the peak antibody response (t_1). After t_1, we model antibody decay using a power function, with the parameters listed below:

Growth phase: $y'(t) = \mu y(t),\ \ t \in [0, t_1]$
Decay phase: $y'(t) = -y_1^r \alpha[1 + (r+1)y_1^{r-1}\alpha(t - t_1)]^{-\frac{r}{r-1}},\ \ t \in [t_1, \infty)$

Parameters:
y_0 = Baseline antibody concentration (U/mL); i.e., $y_0 = y(0)$
y_1 = Peak antibody concentration (U/mL); i.e., $y_1 = y(t_1)$
μ = Antibody growth rate
t_1 = Time to peak antibody concentration (days)
α = Antibody decay rate (1/days)
r = Antibody decay shape

medRxiv preprint doi: https://doi.org/10.1101/2025.06.04.25328941; this version posted August 4, 2025. The copyright holder for this preprint (which was not certified by peer review) is the author/funder, who has granted medRxiv a license to display the preprint in perpetuity.
It is made available under a CC-BY 4.0 International license .

Figure S2.1: Grey dots and lines depict observed longitudinal antibody responses measured from confirmed enteric fever patients, each dot is a measured value and the lines connect individuals. The colored line is the median modeled antibody responses, red indicates higher quantitative antibody responses and blue indicates lower responses. Antibody responses are net (minus) blank and normalized to plate-specific positive controls (Normalized Elisa Units).

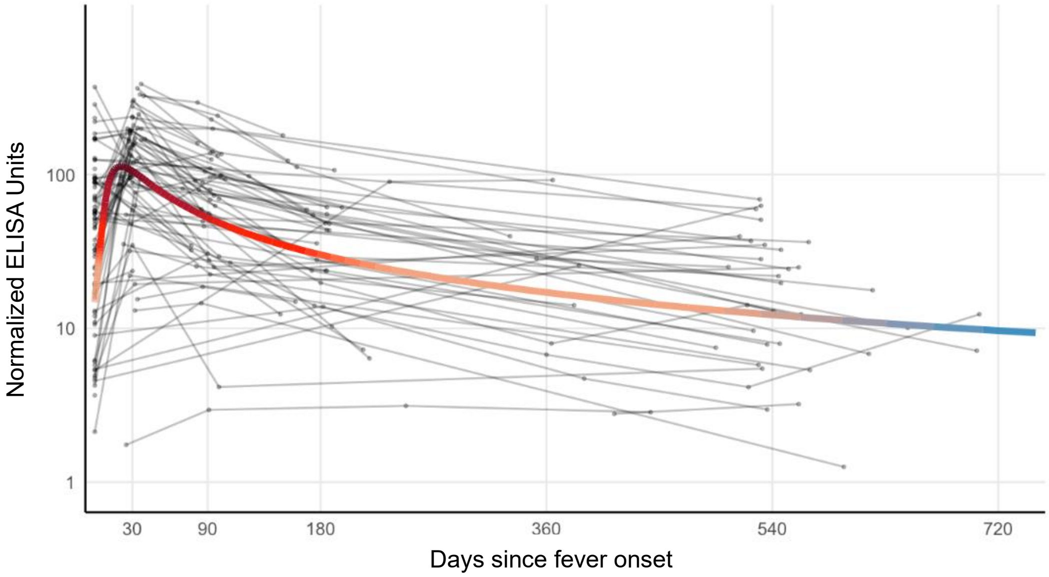

When we subsequently use the fitted seroresponse model to estimate seroincidence from a cross-sectional population survey sample, each participant in the cross-sectional survey contributes a single observation at a random point in the infection process (Figure S2.2). Each individual's seroresponse at that point in time is determined by their particular seroresponse parameters. When computing the seroincidence estimate's standard error, **serocalculator** incorporates both the between-person variation in seroresponse and the estimation error from the pre-estimated longitudinal model.

medRxiv preprint doi: https://doi.org/10.1101/2025.06.04.25328941; this version posted August 4, 2025. The copyright holder for this preprint (which was not certified by peer review) is the author/funder, who has granted medRxiv a license to display the preprint in perpetuity.
It is made available under a CC-BY 4.0 International license .

Figure S2.2: a) Sample cross-sectional population data with low (blue) to high (red) antibody responses. b) Two-phase modeled longitudinal seroresponse curve with colors corresponding to distant vs. recent infection.

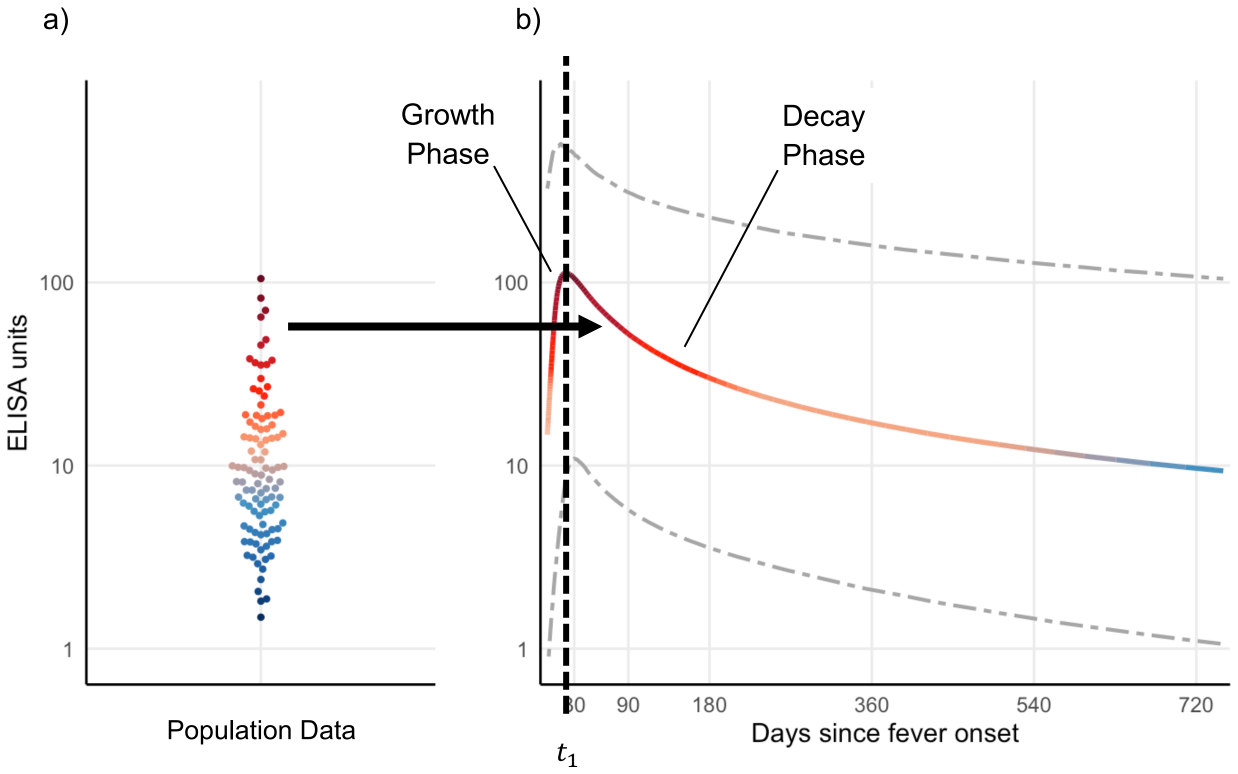

medRxiv preprint doi: https://doi.org/10.1101/2025.06.04.25328941; this version posted August 4, 2025. The copyright holder for this preprint (which was not certified by peer review) is the author/funder, who has granted medRxiv a license to display the preprint in perpetuity.
It is made available under a CC-BY 4.0 International license .

References and Further Reading:

1. de Graaf WF, Kretzschmar MEE, Teunis PFM, Diekmann O. A two-phase within-host model for immune response and its application to serological profiles of pertussis. Epidemics. 2014;9:1-7. doi:10.1016/j.epidem.2014.08.002

2. Teunis PFM, van Eijkeren JCH, de Graaf WF, Marinović AB, Kretzschmar MEE. Linking the seroresponse to infection to within-host heterogeneity in antibody production. Epidemics. 2016;16:33-39. doi:10.1016/j.epidem.2016.04.001

3. Diekmann O, de Graaf WF, Kretzschmar MEE, Teunis PFM. Waning and boosting: on the dynamics of immune status. J Math Biol. 2018;77(6-7):2023-2048. doi:10.1007/s00285-018-1239-5

4. Lotka AJ. Analytical Note on Certain Rhythmic Relations in Organic Systems. Proceedings of the National Academy of Sciences. 1920;6(7):410-415. doi:10.1073/pnas.6.7.410

5. Volterra V. Fluctuations in the Abundance of a Species considered Mathematically. Nature. 1926;118(2972):558-560. doi:10.1038/118558a0

6. Laird NM, Ware JH. Random-Effects Models for Longitudinal Data. Biometrics. 1982;38(4):963. doi:10.2307/2529876

7. Bayes T. LII. An essay towards solving a problem in the doctrine of chances. By the late Rev. Mr. Bayes, F. R. S. communicated by Mr. Price, in a letter to John Canton, A. M. F. R. S. Philos Trans R Soc Lond. 1763;53:370-418. doi:10.1098/rstl.1763.0053

8. Aiemjoy K, Seidman JC, Saha S, et al. Estimating typhoid incidence from community-based serosurveys: a multicohort study. Lancet Microbe. 2022;3(8):e578-e587. doi:10.1016/S2666-5247(22)00114-8

9. Simonsen J, Mølbak K, Falkenhorst G, Krogfelt KA, Linneberg A, Teunis PFM. Estimation of incidences of infectious diseases based on antibody measurements. Stat Med. 2009;28(14):1882-1895. doi:10.1002/sim.3592

medRxiv preprint doi: https://doi.org/10.1101/2025.06.04.25328941; this version posted August 4, 2025. The copyright holder for this preprint (which was not certified by peer review) is the author/funder, who has granted medRxiv a license to display the preprint in perpetuity.
It is made available under a CC-BY 4.0 International license .

S3. Further Details on Noise Parameters

Seroincidence estimates can be biased by both systematic and random error (e.g. noise).[1] **serocalculator** is designed to incorporate two sources of noise: biological noise from cross-reactivity or non-specific binding, and measurement noise during laboratory analysis. In the package, biologic noise is denoted by ν ("nu") and measurement noise is denoted by ε ("epsilon").[1]

The biological noise, ν, represents error from cross-reactivity to other antibodies. Users of **serocalculator** should estimate ν using the 95th percentile of the distribution of antibody responses to the antigen-isotype in a population with no exposure. Biological noise is assumed to be random and is always positive.

Measurement noise, ε, represents laboratory assay variability from the laboratory testing process. Users of **serocalculator** should estimate ε using the CV (coefficient of variation; i.e., the ratio of the standard deviation to the mean) for replicate measurements of the same biological samples.[2] Note that the CV should ideally be estimated using replicates across plates rather than within the same plate. Measurement noise can be positive or negative and can be reduced by running laboratory assays in duplicate and taking the mean.

The full details of these noise parameters were published by Teunis and Eijkeren in 2020 and specific noise estimation procedures were described by Aiemjoy et al. in 2022.[1,2]

References:

1. Teunis PFM, van Eijkeren JCH. Estimation of seroconversion rates for infectious diseases: Effects of age and noise. *Stat Med*. 2020;39(21):2799-2814. doi:10.1002/sim.8578
2. Aiemjoy K, Seidman JC, Saha S, et al. Estimating typhoid incidence from community-based serosurveys: a multicohort study. *Lancet Microbe*. 2022;3(8):e578-e587. doi:10.1016/S2666-5247(22)00114-8

medRxiv preprint doi: https://doi.org/10.1101/2025.06.04.25328941; this version posted August 4, 2025. The copyright holder for this preprint (which was not certified by peer review) is the author/funder, who has granted medRxiv a license to display the preprint in perpetuity.
It is made available under a CC-BY 4.0 International license .

S4. Description of Use Case Dataset

Enteric fever remains a major cause of morbidity and mortality in south Asia, southeast Asia, and sub-Saharan Africa, particularly for children.[1] It is caused by systemic infection with *Salmonella enterica* serovars Typhi and Paratyphi, which typically produce non-specific symptoms of fever, headache, and malaise. Complications can include intestinal perforation, gastrointestinal bleeding, altered mental state, septic shock, and death.[2,3] Although vaccines are available, many countries do not have the evidence of enteric fever burden to justify vaccine introduction. Estimates of seroincidence rates can provide the necessary data for vaccine introductions, while also specifying the population groups that may experience the highest risks of infection.

In the use case, we elect to estimate our seroincidence based on both IgA and IgG antibody responses to the Hemolysin E (HlyE) antigen (in column 'antigen_iso'), which have been shown to reliably distinguish enteric fever from other invasive bacterial infections.[4] Data for the use case comes from the SeroEpidemiology and Environmental Surveillance (SEES) for enteric fever study, which was conducted in Bangladesh, Nepal, and Pakistan.[5] It includes quantitative antibody responses from blood culture-confirmed enteric fever cases from the prospective clinical surveillance study, Surveillance for Enteric Fever in Asia Project (SEAP) that ran from 2016 to 2019 across sites in Bangladesh, Nepal, and Pakistan,[6] as well as population-based cross-sectional serologic samples collected between 2019-2021 from the same catchment areas.[5] The study sought to evaluate whether new diagnostic serological markers for enteric fever could reliably estimate population-level incidence.

In the population-based arm, households were selected using a single-stage, cluster random sampling method.[7] A grid was laid onto a map of each catchment area, dividing each area into 1000-2500 cells. Densely populated areas were further broken down into more cells of smaller size. Cells were then randomly selected from each catchment area and a field team member approached every household in that cell. Individuals were randomly selected using an age-stratified sample (age 0–4 years, age 5–9 years, age 10–15 years, and age 16–25 years). Participants were followed up to three times approximately 6 months apart.

medRxiv preprint doi: https://doi.org/10.1101/2025.06.04.25328941; this version posted August 4, 2025. The copyright holder for this preprint (which was not certified by peer review) is the author/funder, who has granted medRxiv a license to display the preprint in perpetuity.
It is made available under a CC-BY 4.0 International license .

The SEES study demonstrated that when combined with longitudinal antibody dynamics models, cross-sectionally collected antibody responses to HlyE and LPS could reliably estimate seroconversion rates and capture differences in seroconversion that correlated with clinical incidence estimates.[5] The available data from this study include quantitative antibody responses (IgA and IgG) to *S.* Typhi and *S.* Paratyphi antigens HlyE and LPS from both confirmed enteric fever patients and population-based participants from the same catchment areas. Full details of the SEAP and SEES studies have been previously elsewhere.[5-7]

References:

1. Stanaway JD, Reiner RC, Blacker BF, et al. The global burden of typhoid and paratyphoid fevers: a systematic analysis for the Global Burden of Disease Study 2017. Lancet Infect Dis. 2019;19(4):369-381. doi:10.1016/S1473-3099(18)30685-6
2. Pegues D, Miller S. Salmonellosis. In: Loscalzo J, Fauci A, Kasper D, Hauser S, Longo D, Jameson J, eds. Harrison's Principles of Internal Medicine, 21e. 21st ed. McGraw-Hill Education; 2022. Accessed September 17, 2024. https://accessmedicine.mhmedical.com/content.aspx?bookid=3095&sectionid=265422529
3. Parry CM, Hien TT, Dougan G, White NJ, Farrar JJ. Typhoid Fever. New England Journal of Medicine. 2002;347(22):1770-1782. doi:10.1056/NEJMra020201
4. Andrews JR, Khanam F, Rahman N, et al. Plasma Immunoglobulin A Responses Against 2 Salmonella Typhi Antigens Identify Patients With Typhoid Fever. Clinical Infectious Diseases. 2019;68(6):949-955. doi:10.1093/cid/ciy578
5. Aiemjoy K, Seidman JC, Saha S, et al. Estimating typhoid incidence from community-based serosurveys: a multicohort study. Lancet Microbe. 2022;3(8):e578-e587. doi:10.1016/S2666-5247(22)00114-8
6. Garrett DO, Longley AT, Aiemjoy K, et al. Incidence of typhoid and paratyphoid fever in Bangladesh, Nepal, and Pakistan: results of the Surveillance for Enteric Fever in Asia Project. Lancet Glob Health. 2022;10(7):e978-e988. doi:10.1016/S2214-109X(22)00119-X
7. Andrews JR, Vaidya K, Saha S, et al. Healthcare Utilization Patterns for Acute Febrile Illness in Bangladesh, Nepal, and Pakistan: Results from the Surveillance for Enteric Fever in Asia Project. Clin Infect Dis. 2020;71(Suppl 3):S248-S256. doi:10.1093/cid/ciaa1321
